# Neurotrophic Effect of *Citrus* Auraptene: Neuritogenic Activity in PC12 Cells

**DOI:** 10.3390/ijms13055338

**Published:** 2012-04-27

**Authors:** Yoshiko Furukawa, Sono Watanabe, Satoshi Okuyama, Mitsunari Nakajima

**Affiliations:** Department of Pharmaceutical Pharmacology, College of Pharmaceutical Sciences, Matsuyama University, 4-2 Bunkyo-cho, Matsuyama, Ehime 790-8578, Japan; E-Mails: watanabe.sono.mm@ehime-u.ac.jp (S.W.); sokuyama@cc.matsuyama-u.ac.jp (S.O.); mnakajim@cc.matsuyama-u.ac.jp (M.N.)

**Keywords:** auraptene, ERK1/2, PC12 cells, neurons, *Citrus grandis*, neurite outgrowth

## Abstract

The activation of extracellular signal-regulated kinases (ERK) leads to a number of cellular changes associated with the development of long-term memory. Using cultured cortical neurons, we previously showed that the *n*-hexane extract prepared from the peels of *Citrus grandis* (Kawachi bankan) induces the activation of ERK1/2 and that one of the compounds with this ability in the extract is 3,5,6,7,8,3′,4′-heptamethoxyflavone (HMF), a *Citrus* polymethoxyflavone. In fact, we found that HMF has the ability to rescue mice from drug-induced learning impairment. This hexane extract contains auraptene (AUR), a coumarin derivative with a monoterpene unit, together with HMF. The present study was designed to investigate the effect of AUR *in vitro*. Our results show that 1) AUR had the ability to induce the activation of ERK1/2 in not only cortical neurons but also the rat pheochromocytoma cell line (PC12 cells), which is a model system for studies on neuronal proliferation and differentiation; and 2) AUR had the ability to promote neurite outgrowth from PC12 cells.

## 1. Introduction

Extracellular signal-regulated kinases 1/2 (ERK1/2) are components of the mitogen-activated protein kinase (MAPK) signaling cascade. Recent studies have shown that ERK1/2 are involved in synaptic plasticity and in the development of long-term memory in the central nervous system (CNS) [1,2]. In order to search for *Citrus* compounds beneficial for the treatment of neurodegenerative neurological disorders, we previously screened some *Citrus* extracts by evaluating their ability to activate ERK1/2 in cultured cortical neurons [3]. Consequentially, we found that 1) the *n*-hexane extract of *Citrus grandis* (Kawachi bankan) has the ability to activate ERK1/2; 2) this extract contains 3 polymethoxyflavones (3,5,6,7,8,3′,4′-heptamethoxyflavone, HMF; nobiletin [5,6,7,8,3′,4′-hexamethoxy flavone], NBT; and tangeretin [5,6,7,8,4′-pentamethoxyflavone], TGN) and 1 coumarin derivative (auraptene, 7-geranyloxycoumarin; AUR); and 3) one of the active compounds is HMF. In that study we also extensively investigated *in vitro* the ERK1/2-activating effect of HMF in comparison with that of NBT and TGN, and then found that HMF administered to mice treated with NMDA receptor antagonist MK-801 restores the MK-801-induced deterioration of spatial learning performance in the Morris water-maze task. On the other hand, there are few *in vitro* or *in vivo* studies about the effect of AUR on the CNS. In the present study, we thus sought to determine whether AUR, like HMF, would have the ability to activate ERK1/2 in neuronal cells. Here, we successfully showed that AUR could activate (namely phosphorylate) ERK1/2 in not only cultured cortical neurons but also rat PC12 pheochromocytoma cells.

**Figure 1:**
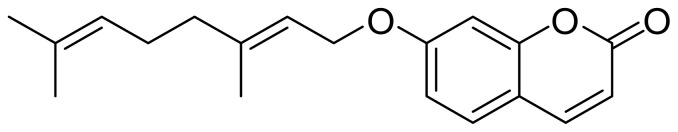
Chemical structure of auraptene (AUR).

AUR is a simple coumarin bearing a geranyloxyl moiety at its C-7 (Figure 1). AUR is a major coumarin of *Citrus* plants, and has been found in not only *C. grandis* (Kawachi bankan) but also various *Citrus* fruits such as grapefruit (*Citrus paradise*) [4]. Numerous studies have indicated that AUR has valuable effects on various biological functions in the peripheral tissues, including anti-inflammatory activity [5,6], anti-carcinogenic activity [7,8], anti-helicobacter activity [9], and regulatory activity on hepatic lipid metabolism [10,11]. On the contrary, there has been only 1 report describing the effect of AUR on the CNS: AUR as well as 7-isopentenyloxycoumarin exerts a protective effect against NMDA-induced excitatory neurotoxicity in mixed cortical cell cultures (composed of neurons and astrocytes) [12]. Excitotoxicity is known to be correlated with various neuronal disorders such as acute brain injury, chronic neurodegeneration (Parkinson’s and Alzheimer’s diseases), epilepsy, spinal cord trauma, ischemic stroke, *etc*. [13], thus suggesting AUR is a novel neuroprotective agent.

In the present study, we found that AUR effectively activated ERK1/2 in PC12 cells. These cells are a useful model system for the study of neuronal differentiation. The exposure of these cells to nerve growth factor (NGF), one of the neurotrophic factors, triggers their differentiation into sympathetic-like neuronal cells [14]. PC12 cells express specific receptor tyrosine kinase (TrkA) on their surface. NGF induces rapid tyrosine phophorylation of TrkA and consequent phosphorylation/activation of signal transduction substrates including ERK1/2 [15,16]. NGF-mediated ERK activation induces phosphorylation of cAMP response element-binding protein (CREB) [17]. Activated CREB then recruits the CREB binding protein (CBP) to the promoter regions of cAMP-responsive genes associated with dendritic spine growth, morphology change, synaptic plasticity and long-term memory [17,18]. Namely, the activation of ERK1/2 by NGF can cause the neurite outgrowth from PC12 cells. Similar to the effect of NGF, various other stimuli such as cyclic AMP, calcium influx [15], depolarization [19], and some phytochemicals such as flavonoids [20] can lead to neurite outgrowth via phosphorylation of ERK. We thus also examined whether or not AUR could stimulate the neuronal differentiation of PC12 cells. Here, we successfully observed that AUR promoted neurite outgrowth from PC12 cells. It is noteworthy that this is the first report on the neurotrophic action of AUR.

## 2. Materials and Methods

### 2.1. Cultures of Rat Neurons

Neurons were prepared from the neocortices of Wistar rats (Nippon SLC, Shizuoka, Japan) at embryonic day 18, as previously described [3]. Neurobasal medium (Invitrogen, Carlsbad, CA, USA) containing B27 supplement (1/50 volume; Invitrogen), glutamine (2 mM; Gibco BRL, Grand Island, NY, USA), penicillin (100 U/mL; Gibco BRL), and streptomycin (100 μg/mL; Gibco BRL) was used for the neuronal cultures. Brain-derived neurotrophic factor (BDNF) was purchased from Pepro Tech. (Rocky Hill, NJ, USA); and AUR, from LKT Lab (St. Paul, MN, USA). HMF was prepared from commercial orange oil (Wako, Osaka, Japan), as previously described [3]. AUR and HMF were dissolved in dimethyl sulfoxide (DMSO) to yield 100 mM stock solutions and diluted to a final concentration of 100 μM for use in the experiments.

### 2.2. Cultures of PC12 Cells

PC12 cells were obtained from Y. Hayashi (Asahikawa Medical College, Asahikawa, Japan). The floating cells were maintained in minimum essential medium (MEM, Gibco BRL) supplemented with 10% horse serum (HS; Sigma-Aldrich, St. Louis, MO, USA), 5% fetal bovine serum (FBS, Gibco BRL), penicillin (100 U/mL), and streptomycin (100 μg/mL). For the experiments, the cells were seeded into culture vessels precoated with poly-l-lysine (Sigma). For evaluation of the neurotrophic effect of NGF and/or test compounds, the culture medium must be shifted to low serum-containing medium to induce transition from the proliferation phase to the differentiation one. The medium was thus shifted to low-serum medium (2% HS and 1% FBS) after an adequate incubation period. Mouse NGF 2.5S (Grade I) was purchased from Alomone Labs Ltd. (Jerusalem, Israel). U0126 and H89 were obtained from Calbiochem Biosciences (San Diego, CA, USA).

### 2.3. Immunoblot Analysis

The cells were seeded in 6-well plates (2 × 10^5^ cells/well), cultured for 48 h in normal-serum medium, and then for 48 h in low-serum medium. The cells were subsequently incubated with test compounds for the desired times. The cell extracts were prepared as previously described [21], and then equal amounts of protein (20 μg) were analyzed by immunoblotting. The antibodies and their sources were the following: rabbit antibody against MAPK 1/2 (Erk1/2-CT), which recognizes the *C*-terminal 35 amino acids of the rat 44-kDa MAPK1/ERK1 and 42-kDa MAPK2/ERK2, from Upstate (Lake Placid, NY, USA); rabbit antibody against phospho-p44/42 MAPK (Thr202/Tyr204), which recognizes phosphorylated ERK1/2 (pERK1/2), rabbit antibodies against CREB and phosphorylated CREB (Ser-133), from Cell Signaling (Woburn, MA, USA); horseradish peroxidase (HRP)-linked anti-rabbit IgG as a secondary antibody, from Cell Signaling. The blots were developed by use of the chemiluminescence method with Plus Western Blotting Detection Reagents (Amersham, Piscataway, NJ, USA).

### 2.4. Assessment of Process Formation

PC12 cells seeded into 24-well plates (1.5 × 10^4^ cells/well) were cultured in normal-serum medium, and then incubated for 24 h in low-serum medium containing test compounds. Neurite outgrowth was determined by manually tracing the length of the longest neurite of each of 50 cells in a field (200× magnification) under observation with a phase-contrast microscope (Biozero, Keyence, Osaka, Japan).

## 3. Results and Discussion

### 3.1. AUR-Induced ERK Activation in Cortical Neurons

**Figure 2:**
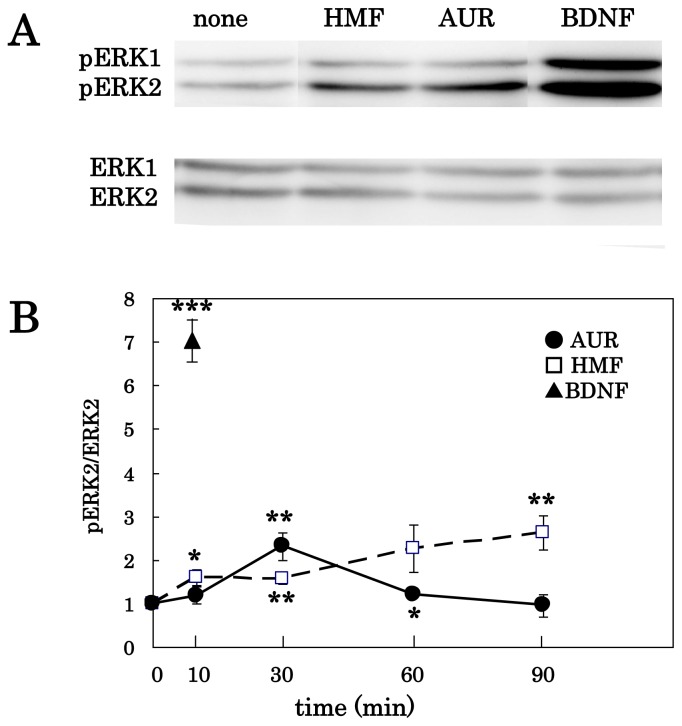
Effects of AUR and 3,5,6,7,8,3′,4′-heptamethoxyflavone (HMF) on extracellular signal-regulated kinases 1/2 (ERK1/2) activation in rat cortical neurons. (**A**) Cells were treated with 100 μM of each *Citrus* compound for 30 min or 50 ng/mL brain-derived neurotrophic factor (BDNF) for 10 min; (**B**) Cells were treated with 100 μM AUR or HMF for various times (0, 10, 30, 60, and 90 min) or with BDNF (50 ng/mL) for 10 min The density ratio of pERK2 to total ERK2 in untreated cultures (time 0) was determined and expressed as 1 arbitrary unit. Results represent mean ± SEM (*n* = 4, different cultures). Significant difference in values between the compound-treated and non-treated cells: ^*^ *P* < 0.05; ^**^ *P* < 0.01; ^***^ *P* < 0.001 (Student’s *t* test).

We previously showed that an *n*-hexane extract (225 mg) of *Citrus grandis* yields AUR (39 mg), HMF (8.5 mg), TGN (2.7 mg), and NBT (1.1 mg) and that HMF, TGN, and NBT have the ability to cause the phosphorylation of ERK1/2 in cultured cortical neurons [3]. Although AUR is the most prevalent component of the *n*-hexane extract of *C. grandis*, its ability to activate ERK had not yet been examined. Therefore, we first tested by immunoblot analysis whether AUR had the ability to promote the phosphorylation of ERK1/2 in cultured neurons. When the cells were treated with 100 μM AUR for 30 min, ERK1/2 were phosphorylated, as was the case with treatment with 100 μM HMF or BDNF at 50 ng/mL (Figure 2A). These results show that AUR as well as HMF and BDNF had the ability to phosphorylate ERK1/2.

We then treated the cells with 100 μM AUR for various periods of times (0, 10, 30, 60, and 90 min). As shown previously, neurons express both ERK1 and ERK2, both of which are phosphorylated in response to BDNF or *Citrus* compounds [3], but only the ERK2 isoform has been suggested to be attributable to neurogenesis and cognitive function [1]. We therefore analyzed the ratio of phosphorylated ERK2 (pERK2) to total ERK2 (ERK2). Figure 2B shows that the AUR-induced phosphorylation of ERK2 in neurons occurred in a time-dependent manner. Enhancement began at 10 min, and a maximal increase was observed at 30 min, which was then followed by a gradual decline (closed circles). In contrast, when the cells were cultured in medium containing 100 μM HMF, the signal was gradually strengthened until at least 90 min after the start of exposure (open squares). BDNF caused rapid phosphorylation of ERK2 within 10 min (closed triangle). These observations indicated that AUR had a unique time-course of ERK1/2-phosphorylation compared to HMF and BDNF. Significant toxicity of AUR was not detected even after incubation for 90 min at a concentration of 100 μM.

### 3.2. AUR-Induced ERK Activation in PC12 Cells

**Figure 3:**
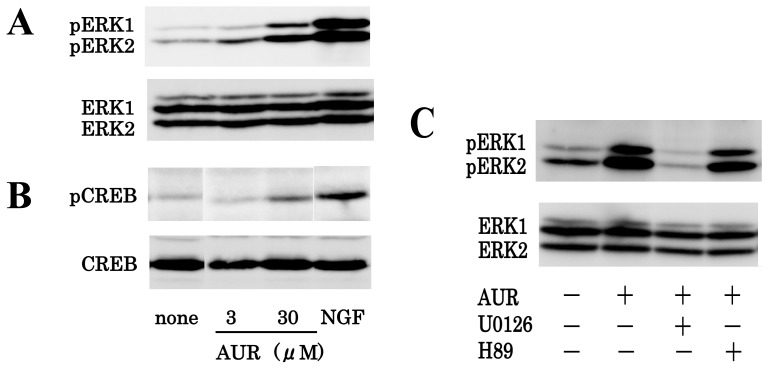
Effects of AUR on ERK1/2 activation and cAMP response element-binding protein (CREB) activation in PC12 cells. (**A**,**B**) Cells were treated with various concentrations (0, 3, and 30 μM) of AUR for 30 min or nerve growth factor (NGF, 50 ng/mL) for 10 min, and cell lysates were then prepared and subjected to immunoblot analysis; (**C**) Cells were left untreated or were pretreated for 30 min with 10 μM U0126, a MEK inhibitor, or with 10 μM H89, a PKA inhibitor, and then incubated for 30 min with 30 μM AUR. Thereafter, cells were subjected to immunoblot analysis.

We then examined the effect of AUR on the phosphorylation of ERK1/2 in PC12 cells, because these cells are often used for studies on neuronal differentiation [22]. Cells were treated with 3 or 30 μM AUR for 30 min or with 50 ng/mL NGF for 10 min. As shown in Figure 3A, AUR increased the phosphorylation of ERK1/2 in PC12 cells (at a concentration of 3 μM ERK1/2 phosphorylation was slightly increased, and at 30 μM strongly increased). NGF, a positive control substance, markedly increased the level of phosphorylated ERK1/2.

ERK1/2 activation is known to lead to a number of cellular changes, such as the expression of CREB before any morphological change [17]. We thus examined the effect of AUR on the phosphorylation of CREB in PC12 cells. As shown in Figure 3B, AUR-induced phosphorylation of CREB correlated well with that of ERK.

It is known that NGF induces the activation (phosphorylation) of MEK (MAPK kinase) 1/2, which then phosphorylates ERK1/2 [22]. Therefore, the effect of a MEK inhibitor (U0126) on AUR-induced phosphorylation of ERK1/2 was next evaluated. As shown in Figure 3C, pretreatment of PC12 cells for 30 min with U0126 abolished the AUR-induced phosphorylation of ERK1/2. U0126 itself had no effect on the basal of ERK1/2-phosphorylation (data not shown). This result indicates that AUR, as well as NGF, could activate MEK1/2, resulting in the phosphorylation of ERK1/2.

Cross talk exists between cAMP-dependent protein kinase A (PKA) and ERK signaling pathways in PC12 cells [23]. The effect of a PKA inhibitor (H89) on AUR-induced phosphorylation of ERK1/2 was next evaluated. As also shown in Figure 3C, the AUR-induced phosphorylation of ERK1/2 was not affected by H89, suggesting that AUR could activate ERK/CREB via a MEK-dependent and PKA-independent pathway.

### 3.3. Effect of AUR on Neuronal Differentiation of PC12 Cells

It is known that neurite outgrowth from PC12 cells is induced via the phosphorylation of ERK1/2 [23]. We thus determined whether AUR could induce the neurite outgrowth from these cells. The extent of PC12 differentiation is typically evaluated by counting the number of cells with extending neurites or by measuring neurite length.

To elucidate the effects of AUR on neurite outgrowth, we incubated PC12 cells on poly-l-lysine-coated culture vessels with various concentrations of AUR (0, 10, 30, and 50 μM) or NGF (50 ng/mL) for 24 h or 48 h, and then measured the length of the longest neurite of each of 50 cells in a phase-contrast microscope field. Our preliminary study indicated that the addition of NGF (30–50 ng/mL) for 24–96 h caused a robust outgrowth of neurite from PC12 cells in a time dependent manner on collagen-coated culture vessels (data not shown) as previously indicated [24]. We used the poly-l-lysine-coated culture vessels in the present study because the PC12 cells were likely to aggregate and come off from the substrates when cells were cultured on collagen-coated culture vessels. When cells were cultured on poly-l-lysine-coated culture vessels, the cells could disperse and attach well, but not extend long and robust neurites.

**Figure 4:**
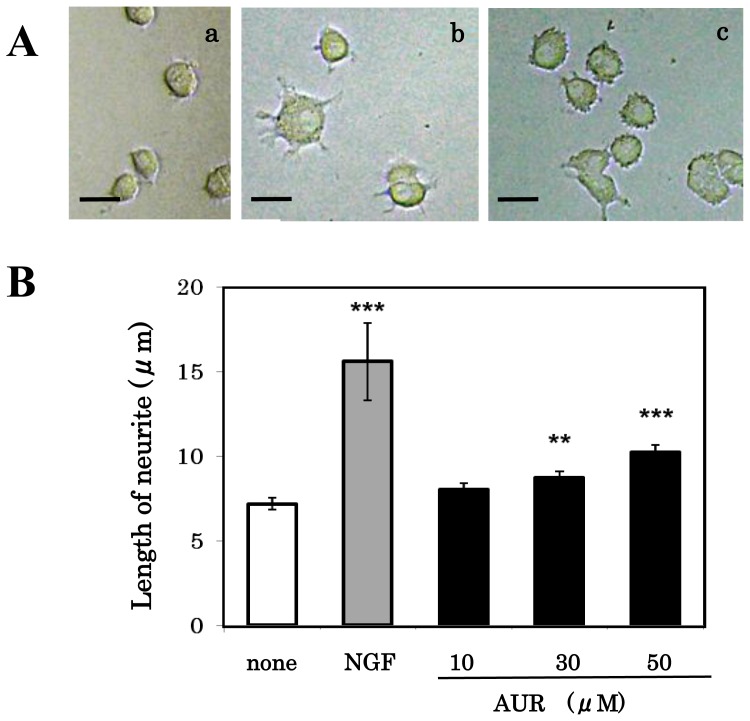
Effects of AUR on neurite outgrowth from PC12 cells. Cells were treated with various concentrations (0, 10, 30, and 50 μM) of AUR or NGF (50 ng/mL) for 24 h, after which phase-contrast photomicrographs of the cells were taken (**A**): **a**, control; **b**, treatment with NGF; **c**, treatment with 30 μM AUR. Scale bar = 20 μm. (**B**): The average radial distance of the longest neurite, measured from the neurite tip to the soma, of 50 cells was determined after 24 h treatment with test drugs. Results represent the mean ± SEM. Significant difference in values between the compound-treated and non-treated cells: ^**^ *P* < 0.01; ^***^ *P* < 0.001 (Student’s *t* test).

As shown in Figure 4, PC12 cells grown in normal medium were round in shape with few neurites (Figure 4A-a and open bar of Figure 4B; 6.69 ± 0.213 μm). NGF treatment resulted in remarkable neurite outgrowth (Figure 4A-b and shaded bar of Figure 4B; 15.6 ± 0.941 μm). Figure 4B shows that AUR treatment for 24 h induced neurite extensions in a dose-dependent manner (closed bars). Figure 4A-c, which is a phase-contrast photomicrograph of PC12 cells after treatment with 30 μM AUR, shows that AUR induced the formation of short neurites (8.74 ± 0.386 μm) by the cells. The neurite length of the cells treated with NGF (50 ng/mL) or AUR (30 μM) for 48 h was 17.0 ± 0.746 μm and 10.0 ± 0.562 μm, respectively, which were a little larger than the levels after 24 h of treatment.

Recent studies on the mechanism of neurite outgrowth from PC12 cells have provided evidence for the roles of a number of different signaling molecular pathways in neuronal differentiation. For example, NBT induces neurite outgrowth of PC12D cells, which were subcloned from the original PC12 cells, by activating a cAMP/PKA/MEK/ERK-dependent pathway [25]. Selenium compounds such as ebselen (2-phenyl-1,2-benzisoselenazol-3(2H)-one), which is artificially synthesized, stimulate neurite outgrowth from PC12 cells via activation of Akt and ERK [26]. Scoparone (6,7-dimethoxycoumarin), which is frequently produced by infected plants, can induce neurite outgrowth from PC12 cells by stimulating the upstream steps of ERK, PKA, protein kinase C (PKC), and Ca^2+^/calmodulin kinase II (CaMK II) [27]. Curcuminoids, the predominant polyphenolic compounds in the rhizome of *Curcuma longa* Linn, promote neurite outgrowth from PC12 cells through ERK- and PKC-dependent pathways [28]. 5-Hydroxy-3,6,7,8,3′,4′-hexamethoxyflavone, an hydroxylated polymethoxyflavone from *Citrus* plants, promotes neurite outgrowth from these cells via the cAMP/PKA/CREB pathway [29]. The AUR-triggered mechanism that underlies the ERK activation in and neuronal differentiation of PC12 cells is not yet clearly understood. In the future, the signaling pathway responsible for the AUR-induced neurite outgrowth from PC12 cells should be investigated.

## 4. Conclusions

This study demonstrates that AUR from *Citrus* plants had the ability to induce activation of ERK1/2 and CREB in cultured neurons and PC12 cells. AUR also had the ability to induce neurite outgrowth from PC12 cells. These results suggest that AUR might be a neurotrophic agent for treating neurodegenerative neurological disorders.
